# Supplementary material for: Prevention of adhesions post-abdominal surgery: Assessing the safety and efficacy of Chitogel with Deferiprone in a rat model
Source: PLoS One. 2021 Jan 14;16(1):e0244503. doi: 10.1371/journal.pone.0244503 (PMC7808615; doi:10.1371/journal.pone.0244503)
Supplement: S1 Table — (DOCX) [file pone.0244503.s001.docx]

**Table of Adhesion Grade with Abrasion alpone and Abrasion with Enterotomy**

| Macroscopic Grading of Adhesion | | | | |
| --- | --- | --- | --- | --- |
| Arm of Study | Treatment | mean | SE | P value |
| Abrasion only | Saline(Control) | 3.98 | 0.33 |  |
|  | Chitogel | 3.51 | 0.29 | 0.20 |
|  | Chitogel + Def 20mM | 3.64 | 0.44 | 0.53 |
|  | Chitogel +Def 10mM | 3.33 | 0.37 | 0.19 |
|  | Chitogel +Def 5mM | 2.77 | 0.29 | 0.001 |
|  | Chitogel +Def 1mM | 2.79 | 0.41 | 0.006 |
|  |  |  |  |  |
| Abrasion with Enterotomy | Saline(Control) | 4.87 | 0.26 |  |
|  | Chitogel | 4.43 | 0.28 | 0.9174 |
|  | Chitogel + Def 5mM | 3.66 | 0.28 | 0.042 |
|  | Chitogel + Def 1mM | 3.69 | 0.32 | 0.018 |
| Microscopic Grading of Fibrosis by Masson’s Trichrome staining | | | | |
|  | Treatment | mean | SE |  |
| Abrasion | Saline(control) | 2.450602 | 0.197389 |  |
|  | Chitogel | 2.161037 | 0.205117 |  |
|  | Chitogel + Def_20mM | 2.085894 | 0.213718 |  |
|  | Chitogel + Def 10mM | 1.854784 | 0.195241 |  |
|  | Chitogel + Def 5mM | 1.819321 | 0.216551 |  |
|  | Chitogel + Def 1mM |  |  |  |
| Abrasion + Enterotomy | _saline | 2.476377 | 0.163667 |  |
|  | cd | 2.562720 | 0.157606 |  |
|  | def_1mM | 2.429625 | 0.173113 |  |
|  | def_5mM | 2.617976 | 0.158781 |  |
