# Supplementary material for: Prevention of adhesions post-abdominal surgery: Assessing the safety and efficacy of Chitogel with Deferiprone in a rat model
Source: PLoS One. 2021 Jan 14;16(1):e0244503. doi: 10.1371/journal.pone.0244503 (PMC7808615; doi:10.1371/journal.pone.0244503)
Supplement: S2 Table — Thin/thick refers to the region of the specimen in which failure occurred. (DOCX) [file pone.0244503.s002.docx]

**Supplementary Table 2:** Number of failures at each site for each treatment group. Thin/thick refers to the region of the specimen in which failure occurred.
