# Supplementary material for: Prevention of adhesions post-abdominal surgery: Assessing the safety and efficacy of Chitogel with Deferiprone in a rat model
Source: PLoS One. 2021 Jan 14;16(1):e0244503. doi: 10.1371/journal.pone.0244503 (PMC7808615; doi:10.1371/journal.pone.0244503)
Supplement: S1 Data — (PDF) [file pone.0244503.s003.pdf]

In [6]:

```
import pandas as pd

df = pd.read_csv("./data/rajan_rat_abdominal_study1_2.tsv", sep="\t")

treatments = df['treatment'].sort_values().unique()

rajan_df = df

rajan_df
```

Out[6]:

	rat_num	markus_score	inflammation_HE	fibrosis_MT	treatment	injury_model
**0**	21	2	2	2	_Saline	abrasion
**1**	22	3	2	2	CD	abrasion
**2**	24	1	3	3	def_20mM	abrasion
**3**	25	2	2	2	def_10mM	abrasion
**4**	26	2	2	2	CD	abrasion
**5**	28	1	3	3	def_20mM	abrasion
**6**	29	0	3	3	def_10mM	abrasion
**7**	30	2	2	2	_Saline	abrasion
**8**	32	1	2	2	def_20mM	abrasion
**9**	33	2	2	2	def_10mM	abrasion
**10**	34	2	1	1	_Saline	abrasion
**11**	35	3	3	3	CD	abrasion
**12**	36	3	2	2	def_20mM	abrasion
**13**	37	2	2	2	def_10mM	abrasion
**14**	38	2	3	3	_Saline	abrasion
**15**	39	1	1	1	CD	abrasion
**16**	41	1	1	1	CD	abrasion
**17**	42	5	3	3	_Saline	abrasion
**18**	43	4	2	2	def_10mM	abrasion
**19**	44	5	3	3	def_20mM	abrasion
**20**	45	5	3	3	def_20mM	abrasion
**21**	46	5	1	1	def_10mM	abrasion
**22**	47	2	2	2	def_10mM	abrasion
**23**	48	1	3	3	_Saline	abrasion
**24**	49	2	1	2	CD	abrasion
**25**	50	5	2	2	def_20mM	abrasion
**26**	51	2	3	3	CD	abrasion
**27**	52	1	3	3	CD	abrasion
**28**	53	4	2	2	def_20mM	abrasion
**29**	54	0	1	1	def_10mM	abrasion
**...**	...	...	...	...	...	...
**78**	109	4	2	2	_Saline	enterotomy
**79**	110	4	2	2	cd	enterotomy
**80**	111	4	2	2	_Saline	enterotomy
**81**	112	3	3	3	cd	enterotomy
**82**	113	5	3	2	_Saline	enterotomy
**83**	114	5	3	3	cd	enterotomy
**84**	115	4	3	3	def_5mM	enterotomy
**85**	116	3	3	3	def_1mM	enterotomy
**86**	117	3	2	2	def_5mM	enterotomy
**87**	118	2	3	3	def_1mM	enterotomy
**88**	119	3	3	3	def_5mM	enterotomy
**89**	120	3	3	3	def_1mM	enterotomy
**90**	121	3	2	2	def_5mM	enterotomy
**91**	122	2	3	3	def_5mM	enterotomy
**92**	123	3	2	2	def_1mM	enterotomy
**93**	124	4	2	2	def_1mM	enterotomy
**94**	125	4	3	3	def_5mM	enterotomy
**95**	126	3	2	2	def_1mM	enterotomy
**96**	127	3	2	2	def_5mM	enterotomy
**97**	128	3	1	1	def_1mM	enterotomy
**98**	129	2	3	3	def_5mM	enterotomy
**99**	130	2	3	3	def_1mM	enterotomy
**100**	131	2	1	1	def_5mM	enterotomy
**101**	132	2	2	2	def_1mM	enterotomy

	rat_num	markus_score	inflammation_HE	fibrosis_MT	treatment	injury_model
**102**	133	3	3	3	def_5mM	enterotomy
**103**	134	2	3	2	def_1mM	enterotomy
**104**	135	2	3	3	def_5mM	enterotomy
**105**	136	3	3	3	def_1mM	enterotomy
**106**	137	3	3	3	def_5mM	enterotomy
**107**	138	2	3	3	def_1mM	enterotomy

108 rows Ã 6 columns

In [7]:

```
%load_ext rpy2.ipython
%R -i treatments
```

```
The rpy2.ipython extension is already loaded. To reload it, use:
  %reload_ext rpy2.ipython
```

In [8]:

```
%%R
library(ordinal)
library(emmeans)
```

In [13]:

```
%%R
rajan_df <- read.csv("./data/rajan_rat_abdominal_study1_2.tsv", sep="\t")
```

Markus (Blinded) Score¶

In [19]:

```
%%R
clm_model <- clm(factor(markus_score, ordered=TRUE, levels = c(0, 1, 2, 3, 4, 5)) ~ treatment + factor(injury_model), data=rajan_df, link=
print(summary(clm_model))
print(emmeans(clm_model,  ~ treatment + injury_model, mode="mean.class", adjust='fdr'))
print(emmeans(clm_model,  pairwise ~ injury_model + treatment, mode="mean.class", adjust='fdr'))
```

```
formula: 
factor(markus_score, ordered = TRUE, levels = c(0, 1, 2, 3, 4, 5)) ~ treatment + factor(injury_model)
data:    rajan_df

 link  threshold nobs logLik  AIC    niter max.grad cond.H 
 logit flexible  108  -170.24 362.48 5(0)  6.66e-08 1.2e+02

Coefficients:
                               Estimate Std. Error z value Pr(>|z|)   
treatmentCD                     -0.6616     0.5245  -1.262  0.20711   
treatmentdef_10mM               -0.9228     0.7125  -1.295  0.19528   
treatmentdef_1mM                -1.7197     0.6363  -2.703  0.00688 **
treatmentdef_20mM               -0.4817     0.7829  -0.615  0.53838   
treatmentdef_5mM                -1.7566     0.5430  -3.235  0.00122 **
factor(injury_model)enterotomy   1.3106     0.4455   2.942  0.00326 **
---
Signif. codes:  0 â€  ***â€   0.001 â€  **â€   0.01 â€  *â€   0.05 â€  .â€   0.1 â€   â€   1

Threshold coefficients:
    Estimate Std. Error z value
0|1  -3.3865     0.6057  -5.591
1|2  -2.1311     0.5104  -4.175
2|3  -0.5379     0.4853  -1.108
3|4   0.6482     0.4932   1.314
4|5   1.7934     0.5140   3.489
 treatment injury_model mean.class    SE  df asymp.LCL asymp.UCL
 _Saline   abrasion           3.98 0.330 Inf      3.33      4.63
 CD        abrasion           3.51 0.289 Inf      2.95      4.08
 def_10mM  abrasion           3.33 0.370 Inf      2.60      4.06
 def_1mM   abrasion           2.79 0.415 Inf      1.98      3.61
 def_20mM  abrasion           3.64 0.443 Inf      2.77      4.51
 def_5mM   abrasion           2.77 0.293 Inf      2.19      3.34
 _Saline   enterotomy         4.87 0.260 Inf      4.36      5.38
 CD        enterotomy         4.43 0.279 Inf      3.88      4.98
 def_10mM  enterotomy         4.25 0.479 Inf      3.31      5.19
 def_1mM   enterotomy         3.69 0.323 Inf      3.06      4.32
 def_20mM  enterotomy         4.55 0.510 Inf      3.55      5.55
 def_5mM   enterotomy         3.66 0.280 Inf      3.12      4.21

Confidence level used: 0.95 
$emmeans
 injury_model treatment mean.class    SE  df asymp.LCL asymp.UCL
 abrasion     _Saline         3.98 0.330 Inf      3.33      4.63
 enterotomy   _Saline         4.87 0.260 Inf      4.36      5.38
 abrasion     CD              3.51 0.289 Inf      2.95      4.08
 enterotomy   CD              4.43 0.279 Inf      3.88      4.98
 abrasion     def_10mM        3.33 0.370 Inf      2.60      4.06
 enterotomy   def_10mM        4.25 0.479 Inf      3.31      5.19
 abrasion     def_1mM         2.79 0.415 Inf      1.98      3.61
 enterotomy   def_1mM         3.69 0.323 Inf      3.06      4.32
 abrasion     def_20mM        3.64 0.443 Inf      2.77      4.51
 enterotomy   def_20mM        4.55 0.510 Inf      3.55      5.55
 abrasion     def_5mM         2.77 0.293 Inf      2.19      3.34
 enterotomy   def_5mM         3.66 0.280 Inf      3.12      4.21

Confidence level used: 0.95 

$contrasts
```

```
 contrast                                  estimate    SE  df z.ratio p.value
 abrasion,_Saline - enterotomy,_Saline      -0.8868 0.293 Inf -3.022  0.0128
 abrasion,_Saline - abrasion,CD              0.4664 0.368 Inf  1.266  0.3476
 abrasion,_Saline - enterotomy,CD           -0.4536 0.491 Inf -0.924  0.5098
 abrasion,_Saline - abrasion,def_10mM        0.6479 0.496 Inf  1.305  0.3476
 abrasion,_Saline - enterotomy,def_10mM     -0.2729 0.671 Inf -0.407  0.7841
 abrasion,_Saline - abrasion,def_1mM         1.1867 0.423 Inf  2.806  0.0207
 abrasion,_Saline - enterotomy,def_1mM       0.2891 0.464 Inf  0.623  0.6817
 abrasion,_Saline - abrasion,def_20mM        0.3402 0.551 Inf  0.617  0.6817
 abrasion,_Saline - enterotomy,def_20mM     -0.5756 0.689 Inf -0.835  0.5671
 abrasion,_Saline - abrasion,def_5mM         1.2109 0.364 Inf  3.324  0.0098
 abrasion,_Saline - enterotomy,def_5mM       0.3151 0.481 Inf  0.655  0.6767
 enterotomy,_Saline - abrasion,CD            1.3532 0.428 Inf  3.160  0.0116
 enterotomy,_Saline - enterotomy,CD          0.4332 0.340 Inf  1.275  0.3476
 enterotomy,_Saline - abrasion,def_10mM      1.5347 0.452 Inf  3.392  0.0092
 enterotomy,_Saline - enterotomy,def_10mM    0.6139 0.484 Inf  1.268  0.3476
 enterotomy,_Saline - abrasion,def_1mM       2.0734 0.546 Inf  3.800  0.0048
 enterotomy,_Saline - enterotomy,def_1mM     1.1759 0.414 Inf  2.840  0.0199
 enterotomy,_Saline - abrasion,def_20mM      1.2269 0.515 Inf  2.383  0.0606
 enterotomy,_Saline - enterotomy,def_20mM    0.3112 0.518 Inf  0.601  0.6824
 enterotomy,_Saline - abrasion,def_5mM       2.0977 0.435 Inf  4.825  0.0001
 enterotomy,_Saline - enterotomy,def_5mM     1.2018 0.348 Inf  3.450  0.0092
 abrasion,CD - enterotomy,CD                -0.9200 0.298 Inf -3.092  0.0116
 abrasion,CD - abrasion,def_10mM             0.1815 0.469 Inf  0.387  0.7841
 abrasion,CD - enterotomy,def_10mM          -0.7393 0.637 Inf -1.161  0.3857
 abrasion,CD - abrasion,def_1mM              0.7203 0.410 Inf  1.755  0.1907
 abrasion,CD - enterotomy,def_1mM           -0.1773 0.434 Inf -0.409  0.7841
 abrasion,CD - abrasion,def_20mM            -0.1262 0.529 Inf -0.239  0.8636
 abrasion,CD - enterotomy,def_20mM          -1.0420 0.657 Inf -1.585  0.2405
 abrasion,CD - abrasion,def_5mM              0.7445 0.341 Inf  2.181  0.0836
 abrasion,CD - enterotomy,def_5mM           -0.1513 0.445 Inf -0.340  0.8072
 enterotomy,CD - abrasion,def_10mM           1.1015 0.463 Inf  2.377  0.0606
 enterotomy,CD - enterotomy,def_10mM         0.1807 0.471 Inf  0.384  0.7841
 enterotomy,CD - abrasion,def_1mM            1.6402 0.573 Inf  2.861  0.0199
 enterotomy,CD - enterotomy,def_1mM          0.7427 0.425 Inf  1.746  0.1907
 enterotomy,CD - abrasion,def_20mM           0.7938 0.525 Inf  1.512  0.2612
 enterotomy,CD - enterotomy,def_20mM        -0.1220 0.506 Inf -0.241  0.8636
 enterotomy,CD - abrasion,def_5mM            1.6645 0.460 Inf  3.616  0.0066
 enterotomy,CD - enterotomy,def_5mM          0.7686 0.350 Inf  2.198  0.0836
 abrasion,def_10mM - enterotomy,def_10mM    -0.9208 0.300 Inf -3.074  0.0116
 abrasion,def_10mM - abrasion,def_1mM        0.5387 0.554 Inf  0.972  0.4965
 abrasion,def_10mM - enterotomy,def_1mM     -0.3588 0.490 Inf -0.732  0.6373
 abrasion,def_10mM - abrasion,def_20mM      -0.3078 0.578 Inf -0.533  0.7132
 abrasion,def_10mM - enterotomy,def_20mM    -1.2235 0.631 Inf -1.941  0.1337
 abrasion,def_10mM - abrasion,def_5mM        0.5630 0.471 Inf  1.195  0.3738
 abrasion,def_10mM - enterotomy,def_5mM     -0.3329 0.464 Inf -0.717  0.6373
 enterotomy,def_10mM - abrasion,def_1mM      1.4596 0.753 Inf  1.938  0.1337
 enterotomy,def_10mM - enterotomy,def_1mM    0.5620 0.576 Inf  0.976  0.4965
 enterotomy,def_10mM - abrasion,def_20mM     0.6131 0.654 Inf  0.937  0.5098
 enterotomy,def_10mM - enterotomy,def_20mM  -0.3027 0.564 Inf -0.537  0.7132
 enterotomy,def_10mM - abrasion,def_5mM      1.4838 0.645 Inf  2.299  0.0700
 enterotomy,def_10mM - enterotomy,def_5mM    0.5879 0.487 Inf  1.208  0.3738
 abrasion,def_1mM - enterotomy,def_1mM      -0.8976 0.283 Inf -3.168  0.0116
 abrasion,def_1mM - abrasion,def_20mM       -0.8465 0.608 Inf -1.391  0.3095
 abrasion,def_1mM - enterotomy,def_20mM     -1.7622 0.771 Inf -2.286  0.0700
 abrasion,def_1mM - abrasion,def_5mM         0.0243 0.399 Inf  0.061  0.9618
 abrasion,def_1mM - enterotomy,def_5mM      -0.8716 0.562 Inf -1.550  0.2498
 enterotomy,def_1mM - abrasion,def_20mM      0.0511 0.550 Inf  0.093  0.9618
 enterotomy,def_1mM - enterotomy,def_20mM   -0.8647 0.604 Inf -1.432  0.2954
 enterotomy,def_1mM - abrasion,def_5mM       0.9218 0.435 Inf  2.121  0.0933
 enterotomy,def_1mM - enterotomy,def_5mM     0.0260 0.427 Inf  0.061  0.9618
 abrasion,def_20mM - enterotomy,def_20mM    -0.9158 0.290 Inf -3.158  0.0116
 abrasion,def_20mM - abrasion,def_5mM        0.8707 0.532 Inf  1.637  0.2236
 abrasion,def_20mM - enterotomy,def_5mM     -0.0251 0.525 Inf -0.048  0.9618
 enterotomy,def_20mM - abrasion,def_5mM      1.7865 0.667 Inf  2.679  0.0286
 enterotomy,def_20mM - enterotomy,def_5mM    0.8906 0.520 Inf  1.712  0.1980
 abrasion,def_5mM - enterotomy,def_5mM      -0.8959 0.290 Inf -3.087  0.0116

P value adjustment: fdr method for 66 tests
```

In [6]:

```
%%R
rajan_df_HE <- subset(rajan_df, !is.na(inflammation_HE))
clm_model <- clm(factor(inflammation_HE, ordered=TRUE) ~ treatment, data=rajan_df_HE, link="logit")
print(summary(clm_model))
print(emmeans(clm_model,  ~ treatment, mode="mean.class", adjust='fdr'))
print(emmeans(clm_model,  pairwise ~ treatment, mode="mean.class", adjust='fdr'))
```

```
formula: factor(inflammation_HE, ordered = TRUE) ~ treatment
data:    rajan_df_HE

 link  threshold nobs logLik AIC    niter max.grad cond.H
 logit flexible  60   -63.47 138.95 4(0)  6.09e-10 4.3e+01

Coefficients:
                  Estimate Std. Error z value Pr(>|z|)
treatmentCD        -0.4210     0.7818  -0.539    0.590
treatmentdef_10mM  -1.1918     0.7511  -1.587    0.113
treatmentdef_20mM  -0.6470     0.7718  -0.838    0.402
treatmentdef_5mM   -1.0778     0.8013  -1.345    0.179

Threshold coefficients:
    Estimate Std. Error z value
1|2 -1.73940    0.60555  -2.872
2|3  0.04147    0.55876   0.074
 treatment mean.class    SE  df asymp.LCL asymp.UCL
 _Saline         2.34 0.209 Inf      1.93      2.75
 CD              2.18 0.222 Inf      1.74      2.61
 def_10mM        1.86 0.200 Inf      1.47      2.25
 def_20mM        2.08 0.219 Inf      1.65      2.51
 def_5mM         1.91 0.233 Inf      1.45      2.36

Confidence level used: 0.95
```

```
$emmeans
 treatment mean.class    SE  df asymp.LCL asymp.UCL
 _Saline         2.34 0.209 Inf      1.93      2.75
 CD              2.18 0.222 Inf      1.74      2.61
 def_10mM        1.86 0.200 Inf      1.47      2.25
 def_20mM        2.08 0.219 Inf      1.65      2.51
 def_5mM         1.91 0.233 Inf      1.45      2.36

Confidence level used: 0.95

$contrasts
 contrast            estimate    SE  df z.ratio p.value
 _Saline - CD          0.1649 0.305 Inf  0.541  0.7357
 _Saline - def_10mM    0.4810 0.290 Inf  1.659  0.7357
 _Saline - def_20mM    0.2570 0.303 Inf  0.849  0.7357
 _Saline - def_5mM     0.4345 0.313 Inf  1.387  0.7357
 CD - def_10mM         0.3161 0.299 Inf  1.056  0.7357
 CD - def_20mM         0.0921 0.312 Inf  0.295  0.8533
 CD - def_5mM          0.2695 0.322 Inf  0.836  0.7357
 def_10mM - def_20mM  -0.2240 0.297 Inf -0.754  0.7357
 def_10mM - def_5mM   -0.0466 0.307 Inf -0.152  0.8796
 def_20mM - def_5mM    0.1774 0.320 Inf  0.554  0.7357

P value adjustment: fdr method for 10 tests
```

In [7]:

```
%%R
rajan_df_MT <- subset(rajan_df, !is.na(fibrosis_MT))
clm_model <- clm(factor(fibrosis_MT, ordered=TRUE) ~ treatment, data=rajan_df_MT, link="logit")
print(summary(clm_model))
print(emmeans(clm_model,  ~ treatment, mode="mean.class", adjust='fdr'))
print(emmeans(clm_model,  pairwise ~ treatment, mode="mean.class", adjust='fdr'))
```

```
formula: factor(fibrosis_MT, ordered = TRUE) ~ treatment
data:    rajan_df_MT

 link  threshold nobs logLik AIC    niter max.grad cond.H
 logit flexible  60   -61.54 135.08 4(0)  1.07e-07 4.2e+01

Coefficients:
                  Estimate Std. Error z value Pr(>|z|)
treatmentCD        -0.7917     0.7932  -0.998   0.3183
treatmentdef_10mM  -1.5763     0.7901  -1.995   0.0460 *
treatmentdef_20mM  -0.9851     0.8066  -1.221   0.2220
treatmentdef_5mM   -1.6682     0.8311  -2.007   0.0447 *
---
Signif. codes:  0 â€ ***â€  0.001 â€ **â€  0.01 â€ *â€  0.05 â€ .â€  0.1 â€  â€  1

Threshold coefficients:
    Estimate Std. Error z value
1|2  -2.2107     0.6553  -3.373
2|3  -0.1982     0.5875  -0.337
 treatment mean.class    SE  df asymp.LCL asymp.UCL
 _Saline         2.45 0.197 Inf      2.06      2.84
 CD              2.16 0.205 Inf      1.76      2.56
 def_10mM        1.85 0.195 Inf      1.47      2.24
 def_20mM        2.09 0.214 Inf      1.67      2.50
 def_5mM         1.82 0.217 Inf      1.39      2.24

Confidence level used: 0.95
$emmeans
 treatment mean.class    SE  df asymp.LCL asymp.UCL
 _Saline         2.45 0.197 Inf      2.06      2.84
 CD              2.16 0.205 Inf      1.76      2.56
 def_10mM        1.85 0.195 Inf      1.47      2.24
 def_20mM        2.09 0.214 Inf      1.67      2.50
 def_5mM         1.82 0.217 Inf      1.39      2.24

Confidence level used: 0.95

$contrasts
 contrast            estimate    SE  df z.ratio p.value
 _Saline - CD          0.2896 0.284 Inf  1.018  0.5142
 _Saline - def_10mM    0.5958 0.278 Inf  2.144  0.1603
 _Saline - def_20mM    0.3647 0.291 Inf  1.254  0.5142
 _Saline - def_5mM     0.6313 0.293 Inf  2.152  0.1603
 CD - def_10mM         0.3063 0.283 Inf  1.081  0.5142
 CD - def_20mM         0.0751 0.296 Inf  0.254  0.8885
 CD - def_5mM          0.3417 0.298 Inf  1.145  0.5142
 def_10mM - def_20mM  -0.2311 0.290 Inf -0.798  0.5310
 def_10mM - def_5mM    0.0355 0.291 Inf  0.122  0.9031
 def_20mM - def_5mM    0.2666 0.304 Inf  0.876  0.5310

P value adjustment: fdr method for 10 tests
```

In [8]:

```
%%R
rajan_df_MT_emmeans <- as.data.frame(emmeans(clm_model,  ~ treatment, mode="mean.class", adjust='fdr'))
rajan_df_MT_emmeans
```

```
  treatment mean.class        SE  df asymp.LCL asymp.UCL
1   _Saline   2.450602 0.1973889 Inf  2.063727  2.837477
2        CD   2.161037 0.2051169 Inf  1.759015  2.563059
3  def_10mM   1.854784 0.1952412 Inf  1.472119  2.237450
4  def_20mM   2.085894 0.2137178 Inf  1.667015  2.504773
5   def_5mM   1.819321 0.2165507 Inf  1.394889  2.243752
```

In [9]:

```
%R -o rajan_df_MT_emmeans
```

```
rajan_df_MT_emmeans = rajan_df_MT_emmeans.reindex([0,1,3,2,4])

rajan_df_MT_emmeans
```

Out[9]:

	treatment	mean.class	SE	df	asymp.LCL	asymp.UCL
**0**	_Saline	2.450602	0.197389	inf	2.063727	2.837477
**1**	CD	2.161037	0.205117	inf	1.759015	2.563059
**3**	def_20mM	2.085894	0.213718	inf	1.667015	2.504773
**2**	def_10mM	1.854784	0.195241	inf	1.472119	2.237450
**4**	def_5mM	1.819321	0.216551	inf	1.394889	2.243752

In [10]:

```
%matplotlib inline
from matplotlib import pyplot

bars = []

#set up colours
cmap_tab20 = pyplot.get_cmap('tab20')
colours = list(cmap_tab20.colors[::2])[:5]
colours = [colours[0], colours[1], colours[3], colours[4], colours[2]]

for i, (index, row) in enumerate(rajan_df_MT_emmeans.iterrows()):
    _means = [row['mean.class']]
    _sems = row['SE']
    print(row['treatment'], _means)
    bar = pyplot.bar([i], _means, width=0.6, color=colours[i])
    bars.append(bar)
    error = pyplot.errorbar([i], _means, yerr=_sems, color='black')

pyplot.xticks([0,1,2,3, 4], ["Saline", 'CD', 'Def_20mM', 'Def_10mM', 'Def_5mM'])
pyplot.ylabel("Grade of fibrosis on Masson's Trichrome\n(mean of the ordinal response)")

pyplot.text(1.5, 2.72, "p=0.046", fontsize=10, horizontalalignment='center')
pyplot.hlines(2.7, 0, 3)

pyplot.text(2, 2.82, "p=0.045", fontsize=10, horizontalalignment='center')
pyplot.hlines(2.8, 0, 4)

pyplot.ylim(1.4, 2.9)

pyplot.savefig("./results/study1_MT_scores.jpg", dpi=300, bbox_inches="tight")
```

```
_Saline [2.4506017628330117]
CD [2.1610368841514256]
def_20mM [2.0858940032142805]
def_10mM [1.854784387246275]
def_5mM [1.819320634114203]
```

In [11]:

```
%matplotlib inline
from matplotlib import pyplot
import seaborn

#set up colours
seaborn.set_palette('tab10')


ax = seaborn.barplot(x="treatment", y="markus_score", data=rajan_df, palette=colours)
ax.set_ylabel("Grade of adhesion\n(mean of the ordinal response)")

#pyplot.text(0.5, 70 +1, "p=0.0398", fontsize=10, horizontalalignment='center')
#pyplot.hlines(70, 0, 1)

#pyplot.text(1,75 +1, "p=0.0096", fontsize=10, horizontalalignment='center')
#pyplot.hlines(75, 0, 2)

#pyplot.ylim(0, 80)
fig = ax.get_figure()
fig.savefig("./results/study1_markus_score.jpg", dpi=300, bbox_inches='tight')
```

Method:¶

All statistics were performed using R statistical software (R Foundation for Statistical Computing, Vienna, Austria) through the Jupyter notebook interface. The R package "ordinal" was used for ordinal regression. The "clm" function was used to fit a Cumulative Link Model, with the semi-quantitative adhesion scores as the ordinal outcome variable. The means of the ordinal response (interpreted as a numeric value from 1 to the number of classes) were calculated and post-hoc pairwise contrasts for each pair of levels of the treatment variable were compared using the "emmeans" package. (cite emmeans)
